# Advances in the Application of Amino Acids in the Preservation of Postharvest Fruits and Vegetables

**DOI:** 10.3390/foods15101650

**Published:** 2026-05-09

**Authors:** Changsong Shang, Lei Ma, Wanyu Yan, Mengyue Jiang, Jiacheng Hu, Dilireba Shataer, Yating Zhao, Yuanyuan Hou

**Affiliations:** 1 College of Smart Agriculture (Research Institute), Xinjiang University, Urumqi 830046, China; shangchangsong@stu.xju.edu.cn (C.S.); 13993939201@163.com (L.M.); yanwanyu0518@163.com (W.Y.); 13150375661@163.com (M.J.); 18903798655@163.com (J.H.); dlrb_shataer2021@xju.edu.cn (D.S.); 2 College of Food Science and Pharmaceutical Science, Xinjiang Agricultural University, Urumqi 830052, China; zhaoyt@xjau.edu.cn

**Keywords:** amino acids, postharvest preservation, fruits and vegetables, biochemical mechanisms, quality deterioration

## Abstract

Postharvest loss of fruits and vegetables remains a significant global challenge, while concerns over chemical residues, energy consumption, and storage-induced physiological disorders have accelerated the search for safe and eco-friendly preservation strategies. As key plant metabolites and signaling molecules, amino acids are gaining more attention for their roles in postharvest biology. This article reviews the primary postharvest issues of fruits and vegetables, the functional roles of representative amino acids, including γ-aminobutyric acid (GABA), glutamate (Glu), arginine (Arg), cysteine (Cys), proline (Pro), and phenylalanine (Phe), with emphasis on the postharvest applications and mechanisms of action of amino acids. Increasing evidence suggests that amino acids can improve postharvest performance mainly by mitigating oxidative stress, enhancing ROS scavenging capacity, retaining cellular membrane integrity, regulating osmotic and energy metabolism, activating phenylpropanoid-mediated defense, and modulating respiration–ethylene processes. These responses contribute to reducing chilling injury, decay, browning, softening, and senescence, thereby helping maintain color, texture, nutritional value, and storage quality. However, the effectiveness of amino acid treatments varies depending on the product types and conditions, and is influenced by concentration, application method, storage environment, and combined preservation strategies. The information in this review provides a systematic foundation for understanding amino acid-mediated modulation mechanisms of postharvest horticultural crops and for developing more environmentally friendly postharvest preservation technologies, while highlighting the need for treatment standardization, mechanistic validation, and commercial-scale evaluation.

## 1. Introduction

Fresh fruits and vegetables are rich in nutrients and provide multiple health benefits, but their postharvest losses worldwide remain at very high levels, generally reaching 30% to 50% of total production [1]. Although traditional preservation methods, such as cold storage, controlled atmospheres, and chemical treatments, can slow down the postharvest deterioration in fruits and vegetables during storage, they possess significant limitations. For example, refrigeration consumes substantial energy and may induce chilling injury in cold-sensitive horticultural products [2]. Controlled-atmosphere storage can delay respiration and ripening by regulating O_2_ and CO_2_ levels, but its dependence on airtight facilities, gas-conditioning systems, refrigeration units, and continuous atmosphere control may increase operational complexity and costs, limiting its adoption in small- and medium-scale supply chains [3]. Synthetic pesticides and fungicides remain important for protecting fruits and vegetables, but recent residue-monitoring studies have reported multiresidue contamination, non-approved pesticide detection, maximum residue limit (MRL) exceedance, and potential acute or cumulative health risks, especially for children, thereby increasing the demand for safer and more eco-friendly preservation strategies [4,5]. Therefore, developing green and natural sustainable preservation technologies has become an urgent problem to be solved in the postharvest fruits and vegetables storage and transportation industry.

Plant amino acids originate from several main pathways branching from central metabolism: the glutamate family amino acids link nitrogen assimilation with stress adaptation [6,7], the aspartate family pathway provides several essential amino acids and related metabolites [8,9], and aromatic amino acids generated by the shikimate pathway provide substrates for phenylpropanoid-derived antioxidants and defense compounds [10,11]. This metabolic pathway is associated with postharvest biology, as the same metabolic branches are linked to key postharvest deterioration processes, including membrane stability, reactive oxygen species (ROS) homeostasis, ethylene-related ripening, phenolic metabolism, and defense activation [12].

Increasing research demonstrates that amino acids play multifaceted roles in the preservation of postharvest fruits and vegetables, including enhancing disease resistance, alleviating chilling injury, maintaining quality, and delaying senescence [12,13,14,15]. For instance, exogenous L-phenylalanine (L-Phe) treatment can induce antioxidant defense responses to mitigate chilling injury in postharvest muskmelons [16], whereas proline (Pro) improves cold tolerance in postharvest pomegranates through the activation of antioxidant activity and phenolic accumulation [17]. L-arginine (L-Arg) and L-methionine (L-Met) can significantly inhibit the growth of gray mold (*Botrytis cinerea*) on postharvest cherry tomato fruits and reduce the incidence of rot [18]. Furthermore, amino acids have been proven to play a role in maintaining quality and delaying senescence in fruits and vegetables [19,20]. However, the current evidence remains fragmented because many studies focus on a single amino acid, crop species, storage condition, or postharvest disorder. It is still unclear which mechanisms are broadly supported, which responses are crop- or condition-specific, and how amino acid treatments compare with or complement existing preservation technologies. This review introduces the biosynthetic sources and signal transduction of amino acids in plants, and explores how these characteristics are relevant to postharvest biology. Furthermore, the applications of amino acids in the preservation of postharvest fruits and vegetables are also summarized, synthesizing recent related research organized around major postharvest issues—disease, chilling injury, quality deterioration, and senescence—with emphasis on the mechanisms of action of amino acids. By integrating functional classification, typical applications, pathways of action, and practical limitations, this review consolidates amino acid classification, signal transduction, and post-harvest applications into a unified mechanistic framework, while critically comparing the strength of evidence, conditional dependencies, and practical applicability across various horticultural products and preservation contexts.

## 2. Main Issues of Fruits and Vegetables

The shelf life of postharvest fruits and vegetables is affected by a series of interconnected physiological variations. Pathogen infection leads to tissue necrosis and redox imbalance [21]; low-temperature stress triggers metabolic disorder and cell membrane damage [22]; the ripening and senescence process inevitably leads to deterioration of quality characteristics, such as texture, color, and nutritional components [23]. In practice, these processes overlap: chilling often induces oxidative stress, disrupting cell membrane structure and accelerating senescence [24], whereas physiological disorder and the senescence process render fruit and vegetable tissues more vulnerable to opportunistic microbes [25]. At the cellular level, membrane lipid peroxidation resulting from excessive accumulation of ROS is considered one of the primary causes of quality deterioration in postharvest horticultural products. For example, chilling-sensitive fruits exhibit dramatic electrolyte leakage, increased malondialdehyde (MDA), and reduced membrane fluidity under cold stress [26,27]. Likewise, infection by necrotrophic fungi (such as *Botrytis cinerea*) elicits a rapid oxidative burst that disrupts host redox balance [28]. In both cases, antioxidant defenses (ascorbate, glutathione, and peroxidases) are repressed, so ROS damage lipids, proteins, and DNA, causing cell death, browning, and softening. Hormonal signaling is also disrupted: chilling stress alters calcium and ethylene homeostasis [26], while ethylene and abscisic acid (ABA) promote the incidence of disease and senescence [29]. Thus, despite their different triggers and specific regulatory mechanisms, chilling injury, disease, quality deterioration, and senescence are related to multiple biochemical processes, such as lipid peroxidation, antioxidant system, enzyme activation (such as lipoxygenase, polyphenol oxidase), sugar and energy metabolism, cell wall degradation, and secondary metabolite biosynthesis, thereby impacting the storage quality and shelf life in postharvest fruits and vegetables.

## 3. Amino Acid Biosynthesis and Signal Transduction in Plants

### 3.1. Amino Acid Biosynthesis

#### 3.1.1. Signaling Molecules: Glu, GABA, Arg

In plants, Glu is primarily synthesized through the glutamine synthetase (GS)/glutamate synthase (GOGAT) cycle, in which ammonia is first incorporated into glutamine (Gln) by GS, and then transferred to 2-oxoglutarate by GOGAT [30]. Therefore, Glu and Gln serve as central nitrogen donors in plants, participating in the biosynthesis of nearly all other amino acids [31]. For example, Glu is decarboxylated by glutamate decarboxylase (GAD) to form GABA [32], and it can also be aminated to glutamate-5-semialdehyde, which is subsequently converted to ornithine and ultimately synthesized into Arg [33]. These “Glu-family” amino acids not only participate in protein synthesis but also possess multiple physiological functions. In particular, GABA is a stress-responsive metabolite positioned at the interface of primary carbon–nitrogen metabolism and adaptive regulation [34]. It is generated by the irreversible decarboxylation of Glu by glutamate decarboxylase and is catabolized through GABA transaminase and succinic semialdehyde dehydrogenase to re-enter the TCA cycle [7]. GABA elevation can modulate anion transport and guard-cell behavior via aluminum-activated malate transporters (ALMTs) and is associated with antioxidant capacity, ionic balance, and pH homeostasis during stress [35]. With the highest nitrogen-to-carbon ratio among proteinogenic amino acids, Arg serves as an efficient storage form of organic nitrogen in many plant tissues [36]. In plants, Arg is synthesized from Glu via ornithine and citrulline, with the core biosynthetic steps occurring primarily in plastids, consistent with the role of plastids as the main site of basic amino acid production [37]. Beyond nitrogen storage, Arg participates in polyamine biosynthesis through a distinct decarboxylation pathway [38], and polyamines are closely associated with growth regulation, stress, and defense signaling [39]. Arg has also been discussed as a potential source of nitric oxide; however, nitric oxide production in plants involves multiple pathways, and whether arginine-dependent Nitric Oxide Synthase (NOS)-like mechanisms exist in higher plants and their contribution remains controversial [40].

#### 3.1.2. Precursors of Bioactive Compounds: Phe, Cys

Phe is primarily synthesized in plastids via the shikimate pathway, which converts phosphoenolpyruvate and erythrose-4-phosphate into chorismate, and Phe is then formed from chorismate through the prephenate branch predominantly via the arogenate route [41,42]. Phe is the entry point to the phenylpropanoid pathway: phenylalanine ammonia-lyase (PAL) deaminates Phe to cinnamate, which yields a vast array of phenylpropanoids (such as flavonoids, lignin, coumarins, stilbenes) [43]. Over 8000 phenylpropanoid compounds ultimately derive from Phe [44]. These metabolites form the material basis for plant tissue structural integrity and defense responses [45]. Lignin polymers can strengthen cell wall structures, forming protective barriers (Casparian strip, wound-induced lignification), which serve to limit pathogen invasion [46]. Flavonoids and other phenolic compounds (anthocyanins, quercetin, stilbenes, coumarins) function as UV barriers and antioxidants, and many function as phytoalexins against microorganisms [47]. Therefore, phenylpropanoid metabolism based on phenylalanine is a key process determining the postharvest physiological characteristics of fruits and vegetables.

Cys is another important precursor of bioactive compounds. In plants, sulfate is reduced to sulfide [48]. Subsequently, the sulfide reacts with O-acetylserine to produce Cys under the action of O-acetylserine(thiol)lyase [49]. Cys is the first organic product of sulfate assimilation and serves as the precursor for glutathione (GSH) biosynthesis via γ-glutamylcysteine synthetase (γ-ECS) and glutathione synthetase (GSHS), while also supplying reduced sulfur for downstream metabolites [50]. Because GSH is a central component of the AsA–GSH cycle, Cys metabolism is closely linked to ROS detoxification, thiol redox homeostasis, and stress tolerance in harvested tissues [51]. Furthermore, Cys can participate in hydrogen sulfide (H_2_S)-related signaling, further linking sulfur metabolism to plant stress resistance [52].

#### 3.1.3. Osmoprotectants: Pro

Pro is mainly synthesized from Glu through the pyrroline-5-carboxylate synthetase (P5CS) and pyrroline-5-carboxylate reductase pathways [53]. Unlike Glu, GABA, and Arg, Pro mainly functions as an osmoprotectant and redox stabilizer. Its accumulation contributes to osmotic adjustment, membrane stabilization, protein protection, and ROS buffering under storage-related stress [54]. In postharvest fruits and vegetables, direct exogenous Pro application has been shown to reduce electrolyte leakage, MDA and H_2_O_2_ accumulation, enhance antioxidant enzyme activities, and maintain membrane integrity, thereby improving tolerance to chilling-induced oxidative stress [52]. By contrast, when Pro accumulation is induced by H_2_S, ABA, NO, or other upstream stress signals, Pro should be considered mainly as a downstream protective effector involved in osmotic adjustment, redox buffering, and membrane stabilization, rather than as the primary preservative factor [55].

### 3.2. Amino Acid Signaling Pathways

Beyond their metabolic functions, amino acids themselves act as signals that integrate nutritional and stress information [56]. Extracellular Glu can function as a damage-associated cue, as plant-encoded glutamate receptor–like (GLR) ion channels can be gated by amino acids and participate in Ca^2+^ signaling during stress responses [57]. Upon wounding, Glu is released locally and can activate GLR-dependent long-distance Ca^2+^ waves that propagate from the injury site to distant tissues, contributing to systemic signal transduction [58].

At the intracellular level, amino acid status is also linked with energy-sensing pathways that may influence stress adaptation during storage [59]. The target of rapamycin (TOR) serves as a central growth hub that integrates carbon and nitrogen signals to coordinate anabolic processes and developmental progression [60]. In Arabidopsis, nitrate, ammonium, and several proteinogenic amino acids activate TOR through the guanosine triphosphatase *ROP2*, placing diverse nitrogen forms on a shared upstream route [61]. GCN2 functions as a stress-responsive translation regulator that senses amino-acid limitation via uncharged tRNAs and inhibits translation initiation by phosphorylating *eIF2α* [62].

Amino acids participate in signaling layers that intersect with phytohormone pathways, so metabolic shifts in amino acid pools can be translated into hormone-mediated stress outputs [63]. Among these, GABA is frequently discussed as a node that interacts with responses related to ABA, ethylene, and salicylic acid in the context of both abiotic and biotic stress [64]. Arg-centered metabolism is closely linked to polyamine production, and polyamines act as bona fide regulators of development as well as stress and defense signaling rather than being passive end-products [39]. Nitric oxide (NO) is another major signal integrated into stress physiology and hormone crosstalk. However, the contribution of an Arg-dependent NOS-like route in higher plants remains debated and should be considered only one of several proposed NO-generating pathways [65]. Sulfur-related signaling adds a regulatory layer, with H_2_S signaling primarily transmitted through Cys persulfidation, a reversible modification that reprograms protein function and stress adaptation [66].

**Figure 1:**
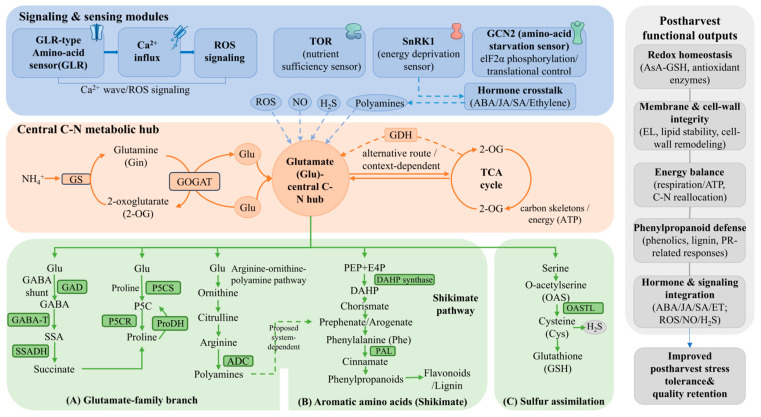
Integration of amino acid biosynthesis and signaling transduction in plants.

Collectively, these signaling pathways ensure that amino acid metabolism is coordinated with environmental challenges (as shown in Figure 1). GLR and Ca^2+^ signals allow wounded or stressed tissues to warn distal parts [58]. Nutrient sensors (TOR, GCN2) adjust metabolism during starvation or abundance [61,67]. Amino acid-derived messengers (GABA, polyamines, NO, H_2_S) fine-tune hormone networks, antioxidant defense systems, and gene expression [68,69].

## 4. Application and Mechanism of Amino Acids in Preservation of Postharvest Fruits and Vegetables

**Figure 2:**
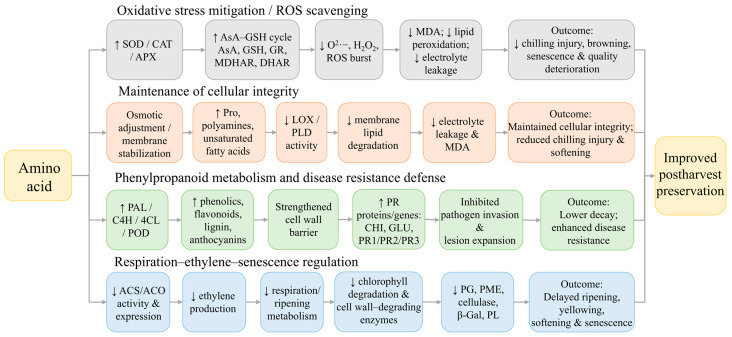
The main mechanistic framework of amino acids in the preservation of postharvest fruits and vegetables.

**Figure 3:**
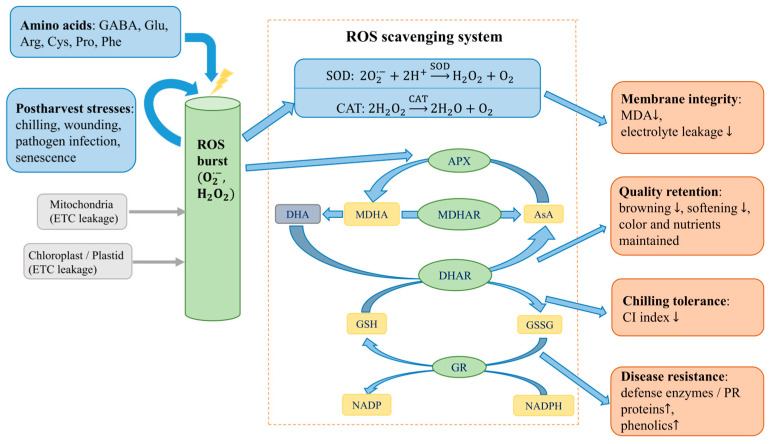
Amino acid–mediated antioxidant defense and ROS scavenging in postharvest fruits and vegetables.

Although different amino acids differ in biosynthetic origin and dominant function, their postharvest effects converge on several recurring mechanisms (as shown in Figure 2). They mainly act by enhancing antioxidant and redox systems (as shown in Figure 3), stabilizing membrane and cellular integrity, activating phenylpropanoid and defense-related metabolism, and modulating respiration–ethylene–senescence processes. These responses help reduce chilling injury, decay, browning, softening, and overall quality decline. However, the magnitude of these effects depends on the amino acid, commodity, treatment method, and storage conditions. The following sections discuss these mechanisms in relation to representative amino acids and their specific postharvest applications.

### 4.1. Application and Mechanism of GABA in the Preservation of Fruits and Vegetables

#### 4.1.1. Disease Control and Mechanisms

GABA is a non-protein amino acid that functions as an endogenous stress signal and can enhance the disease resistance of postharvest horticultural products. Preliminary studies have shown that exogenous GABA treatment can induce resistance to multiple postharvest pathogens in fruits such as tomatoes, pears, and citrus [70,71,72]. Wang et al. [72] found that hypotonic treatment with 10 mM GABA significantly inhibited gray mold (*Botrytis cinerea*), reducing disease incidence and lesion size in harvested cherry tomato fruit stored at 25 °C. Studies have found that GABA-treated tomatoes exhibit a transient NO burst before infection, while S-nitrosoglutathione reductase (GSNOR) activity and gene expression are enhanced to maintain NO stability, keeping NO at optimal defense levels.

Beyond NO signaling, GABA activates other defense mechanisms. Wang et al. [73] soaked fresh-cut *Mesembryanthemum crystallinum* L. in a 200 mg/L GABA solution for 10 min, then stored it at 18 °C for 4 days, and found that this treatment alleviated spoilage caused by *Fusarium*. GABA improved redox homeostasis by enhancing antioxidant capacity and reducing oxidative damage. Transcript enrichment further suggested activation of the salicylic acid pathway and phenylpropanoid metabolism, consistent with stronger phenolic-based antimicrobial defense. Meanwhile, GABA increased the activities of pathogenesis-related protein (PR) antifungal enzymes, namely chitinase (CHI) and β-1,3-glucanase (GLU), reinforcing the enzymatic barrier and limiting the spread of pathogens during storage. Similarly, Chen et al. [74] noted that GABA activated PAL, cinnamate-4-hydroxylase (C4H), and 4-coumarate-CoA ligase (4CL), promoted the accumulation of phenolics, flavonoids, and lignin, and enhanced PR-protein defenses (including GLU and CHI activities), contributing to improving the resistance to *Colletotrichum gloeosporioides* of mango fruit.

#### 4.1.2. Chilling Injury Alleviation and Mechanisms

GABA treatment enhances cold tolerance in fruits and vegetables by activating the antioxidant system. For example, Fan et al. [75] demonstrated that postharvest immersion in 1 mM GABA for 10 min significantly reduced chilling injury symptoms in Chinese olive fruits stored at 2 °C for 100 d by inhibiting O_2_·^−^ and MDA accumulation, which were attributed to the higher superoxide dismutase (SOD), catalase (CAT), and ascorbate peroxidase (APX) activities and GSH and ascorbic acid (AsA) levels in GABA-treated fruits. In kiwifruit, Liu et al. [76] demonstrated that 10 mM GABA immersion for 10 min induced cold tolerance in kiwifruit by increasing the transcription level of *AcMDHAR*, suppressing the expression of *AcAO* (actinidia chinensis ascorbate oxidase), and thereby elevating the AsA content. In aonla fruit, Ali et al. [77] reported that soaking in 5 mM GABA significantly reduced the development of chilling injury. Mechanistically, GABA alleviated chilling injury in aonla fruits by activating the GABA shunt pathway, enhancing the activities of antioxidant enzymes (SOD, CAT, APX, POD), promoting the accumulation of proline and phenolic compounds, and maintaining membrane integrity during cold storage. Furthermore, exogenous GABA significantly up-regulated genes involved in the GABA shunt and polyamine pathways in peaches. Song et al. [78] found that soaking peach fruit in 5 mM GABA induced the GABA biosynthesis genes *PpGAD*, *PpADC*, and the polyamine synthesis gene *PpODC*, while down-regulating the polyamine degradation gene *PpPAO*, promoting the accumulation of putrescine, spermidine, and spermine in peach fruit. Simultaneously, GABA enhanced the expression of the Pro biosynthesis genes *PpP5CS* and *PpOAT*, linking metabolic activity in the GABA pathway to Pro accumulation. These coordinated gene expression changes indicate that GABA enhances cold tolerance in peach fruits by interacting with carbon and nitrogen metabolism. The synergistic interaction between hormones further enhances the protective function of GABA. 

Beyond single-compound treatments, GABA has also shown value in integrated preservation strategies under cold storage. Niazi et al. [79] reported that the combined treatment of GABA and H_2_S reduced chilling injury in persimmon fruits by enhancing antioxidant enzyme activities (SOD, CAT, APX), reducing H_2_O_2_ accumulation, maintaining membrane integrity, promoting the accumulation of phenolic compounds through PAL activation, and inhibiting cell wall-degrading enzymes (polygalacturonase (PG) and methylesterase (PME)) to preserve firmness. In Orlando tangelo fruits, combined GABA and methyl jasmonate treatments synergistically increased juice phenolics and flavonoids, enhanced CAT and POD activities, and reduced MDA levels and cold damage symptoms [80]. Collectively, these mechanistic studies show that GABA integrates metabolic, redox, and hormonal signaling pathways via modulating gene expression and epigenetic control to mitigate chilling injury in postharvest fruits and vegetables. However, there remains a discrepancy in the strength of evidence between single GABA treatment and combined treatment studies.

#### 4.1.3. Quality Maintenance and Mechanisms

GABA has demonstrated the ability to maintain quality in the preservation of postharvest fruits and vegetables [81]. In fresh-cut products, Gao et al. [82] reported that 20 g/L GABA treatment for 10 min significantly reduced the browning index of fresh-cut potatoes by inhibiting polyphenol oxidase (PPO) activity and enhancing antioxidant enzyme activities, thereby limiting oxidative damage and browning development. In fresh-cut stem lettuce, Ru et al. [83] found that 10 mM GABA immersion suppressed enzymatic browning by decreasing PPO activity and down-regulating *LsPPO* expression, as well as by strengthening ROS scavenging ability to maintain membrane stability. In the “Golden Delicious” apples, Li et al. [84] reported that GABA treatment maintained higher pericarp firmness and soluble solid content during storage. Mechanistically, this effect was associated with the accumulation of putrescine, spermidine, and spermine, together with increased endogenous GABA, Glu, and pyruvate in the exocarp, suggesting that GABA helped preserve surface tissue quality by coordinating polyamine metabolism and the GABA shunt.

### 4.2. Application and Mechanism of Glu in the Preservation of Fruits and Vegetables

#### 4.2.1. Disease Control and Mechanisms

Exogenous Glu has become a safe elicitor that suppresses postharvest fungal diseases by stimulating host immunity. In tomato fruit, Sun et al. [85] showed that a brief dip in 100 ppm L-Glu significantly reduced *Botrytis cinerea* decay, and this protection was associated with induction of *GLR* genes, significant up-regulation of *PR* genes (such as *PR1*, *PR2*, *PR3*), and accumulation of defense-related amino acids after treatment. In another study, Yang et al. [86] reported that L-Glu reduced *Alternaria alternata* incidence only when applied sufficiently early before inoculation in tomato fruit. This protective effect was accompanied by the rapid activation of primary nitrogen metabolism (especially the GS/GOGAT cycle) as well as engagement of the GABA bypass, glycolysis, the TCA cycle, and salicylic acid–associated defense signaling pathways. In pear wounds, Jin et al. [15] demonstrated that application of 1.00 mM L-Glu conferred strong resistance to *Penicillium expansum*. It improved GLU, CHI, PAL, POD, and PPO activities, induces the expression of *PR* genes, and promotes the accumulation of Glu, GABA, and Arg. In apple fruit, Yang et al. [87] found that L-Glu inhibited disease development caused by *P. expansum* via increasing total nitrogen, stimulating GS, GOGAT, and glutamate dehydrogenase (GDH) activities, and accelerating consumption of TCA intermediates with higher malate dehydrogenase (MDH) and succinate dehydrogenase (SDH) activities.

#### 4.2.2. Chilling Injury Alleviation and Mechanisms

Research on Glu in alleviating chilling injury in fruits and vegetables is relatively limited, with existing studies primarily focusing on its effects on quality maintenance and disease control. In prunes, Hou et al. [88] revealed that L-Glu mitigated chilling injury primarily through a GLR-triggered Ca^2+^ signal, which further modulated ROS homeostasis, GABA shunt, and energy status. L-Glu up-regulated *PdGLRs* and enhanced cytoplasmic Ca^2+^ concentration alongside calmodulin (CaM) and calmodulin-like protein (CML) accumulation, which was linked to activating antioxidant capacity and reducing lipid peroxidation. This indicated that the induction of Ca^2+^ signaling by L-Glu promoted the GABA shunt and stabilized energy status, helping suppress ROS accumulation, thereby alleviating internal browning of prune fruit during cold storage. Multi-omics analysis further indicated that L-Glu upregulated structural genes such as *PdP5CS*, *PdGAD1*, *Pd4CL*, and *PdUGT*, and promoted the accumulation of Pro, GABA, and phenolic acids. Weighted correlation network analysis (WGCNA) showed that these genes are highly correlated with physiological indicators of chilling injury and are co-regulated by transcription factors such as PdMYB, PdbHLH, and PdWRKY. In summary, multi-omics evidence suggested that L-Glu alleviated chilling injury in prune fruit by synergistically activating amino acid and phenolic metabolic pathways, thereby enhancing antioxidant capacity [89].

#### 4.2.3. Quality Maintenance and Mechanisms

Exogenous Glu maintains postharvest quality mainly by stabilizing carbon allocation, preserving structural integrity, and limiting oxidative deterioration. Jin et al. [90] reported that L-Glu immersion maintained the quality of “Zaosu” pears by inhibiting respiration rate and ethylene production and reducing several deterioration-linked pathways. Mechanistically, L-Glu inhibited chlorophyll degradation by down-regulating *PbSGR1/2*, *PbCHL*, *PbPPH*, *PbRCCR*, and *PbNYC* gene expression. It also prevented softening by inhibiting pectin and cellulose degradation enzymes, as well as the expression of *PbPL* and *Pbβ*-*gal* genes. Furthermore, L-Glu promoted polyamine and GABA accumulation by triggering the activities of arginine decarboxylase (ADC), ornithine decarboxylase (ODC), s-adenosylmethionine ecarboxylase (SAMDC), and the GABA-shunt enzymes (GAD, GABA-T), while repressed polyamine oxidase (PAO), diamine oxidase (DAO) activities, and several genes (such as *PbSSADH*, *PbGDH*, *PbGOGAT*), ultimately increasing endogenous GABA, Glu, and pyruvate. Similarly, Li et al. [91] showed that L-Glu preserved “Qiujin” apple fruit quality by coordinating sucrose–sorbitol metabolism and repressing carotenoid accumulation by down-regulating multiple carotenoid-biosynthetic genes.

In fresh-cut products, Song et al. [92] proved that Glu directly constrained enzymatic browning in fresh-cut potatoes by suppressing PPO activity through acidification and Cu^2+^ chelation, and molecular docking analyses supported a stable interaction of Glu with the PPO active-site region. In parallel, Glu reduced total phenols, maintained the levels of Cys, isoleucine, tyrosine, Phe, and Pro, and increased the levels of Glu, histidine, Arg, glycine, serine, valine, leucine, lysine, Met, and threonine, collectively reducing browning development. By contrast, Qiao et al. [93] used a whole-tuber Glu induction strategy before cutting and showed that Glu also significantly reduced browning and improved sensory quality in fresh-cut potatoes. This effect is closely associated with the accumulation of jasmonic acid (JA) and jasmonic acid-isoleucine (JA-Ile), the up-regulation of JA pathway components (*OPR3*, *JAR1*, *COI1*), enhanced phenylpropanoid metabolism, maintained ROS homeostasis, and reduced microbial infection. Thus, these two studies suggest that Glu can alleviate browning in fresh-cut potatoes through two partially distinct routes: direct physicochemical inhibition of PPO after cutting, or pre-cut signaling induction in whole tubers. Therefore, the mechanistic difference is more likely treatment-dependent than inconsistent.

### 4.3. Application and Mechanism of Arg in the Preservation of Fruits and Vegetables

#### 4.3.1. Disease Control and Mechanisms

Exogenous Arg shows promising potential as a safe inducer for suppressing postharvest fungal diseases, but its effectiveness depends on the specific context. In winter jujubes, 200 μM Arg immersion significantly reduced lesion expansion caused by *Alternaria alternata* infection. Mechanistically, Arg inhibited O_2_·^−^ production and H_2_O_2_ accumulation, which were attributed to enhanced antioxidant enzymes (SOD, CAT, POD, APX) activities; it also enhanced the defense function of disease-related proteins by increasing CHI and GLU activity, and promoted phenylpropanoid metabolism, thereby comprehensively reinforcing redox homeostasis and biochemical barriers against pathogen invasion [94]. Similarly, Wang et al. [95] found that L-Arg reduced *Alternaria* rot in blueberries by enhancing antioxidant capacity, inducing PR proteins (CHI, GLU), activating JA biosynthesis and signaling genes (such as *LOX*, *AOS*, *AOC*, *OPR*, *MYC2*, and *COI1*). In strawberries, immersion in 1 mM L-Arg reduced fruit rot via activating the nitric oxide synthase (NOS) pathway. NO accumulation correlated with increased antioxidant enzymes and decreased lipid peroxidation, and up-regulated expression of *PR1* and the defense enzymes PAL, CHI, GLU, and PPO [96].

In addition to stimulating host defenses, Arg can also act directly on pathogens: against *Botrytis cinerea*, L-Arg inhibited mycelial growth, spore germination, and disrupted membrane integrity, reducing gray mold in vivo [18]. In kiwifruit, L-Arg (5 mM) enhanced *Alternaria alternata* pathogenicity by promoting spore germination and lesion expansion, linked to increased NO and polyamines, ROS generation, and activation of cell wall-degrading enzymes [97]. This indicates that the effectiveness of Arg depends on the treatment concentration, host species, and the stage of pathogen infection, thus requiring validation within specific preservation systems.

#### 4.3.2. Chilling Injury Alleviation and Mechanisms

Exogenous Arg primarily alleviates chilling injury symptoms in fruits and vegetables by maintaining cell membrane integrity and inhibiting oxidative damage. Babalar et al. [98] demonstrated that 1 mM Arg reduced peel browning in pomegranate fruit by enhancing antioxidant enzyme activities (SOD, CAT, and APX), reducing H_2_O_2_, electrolyte leakage (EL), and MDA accumulation. Arg also modulates the balance between phenolic compounds accumulation and browning: PAL activity increases while PPO activity decreases, resulting in a higher PAL to PPO ratio, which favors the accumulation of phenols and anthocyanins and enhances antioxidant capacity, thereby suppressing oxidative browning in pomegranate fruit under low-temperature stress. In cucumbers, immersion in 0.5 mM L-Arg inhibited the increases in chilling injury index and EL during cold storage. Mechanistically, NO biosynthesis driven by L-Arg serves as a key upstream signal to suppress ROS accumulation and cell membrane damage during low-temperature storage [99]. Mahmoudi et al. [100] reported an enhanced strategy in which chitosan nanoparticles loaded with Arg (CTS–Arg NPs) effectively alleviated chilling injury in plums by increasing Pro, reducing EL and MDA, inhibiting PPO, enhancing PAL-related phenol accumulation, improving CAT, POD, APX, and SOD activities, and reducing H_2_O_2_ levels.

#### 4.3.3. Quality Maintenance and Mechanisms

Exogenous Arg has shown potential in maintaining quality in postharvest fruits and vegetables. Pakkish and Mohammadrezakhani [101] reported that pre-harvest spraying of Arg in sweet cherry reduced EL, ROS production, and lipid peroxidation, while increasing the activities of APX, CAT, and SOD, thereby maintaining fruit quality during storage. In postharvest persimmons, L-Arg (1 mM) reduced weight loss and maintained higher firmness, while simultaneously repressing EL, MDA, and H_2_O_2_ and enhancing antioxidant enzyme activity, ascorbic acid, and total phenolic content. Importantly, Arg also inhibited cell wall hydrolytic enzymes (PME, PG, and cellulase) in persimmon fruit during storage, contributing to the maintenance of fruit texture [102]. In fresh-cut pears and apples, Olgaç et al. [103] found that Arg soaking delayed enzymatic browning, maintained lighter color and favorable color values, with reduced browning index, but had limited effects on TSS and firmness, implying that mechanisms of Arg centered on browning control rather than ripeness regulation.

#### 4.3.4. Delaying Senescence and Mechanisms

Arg can delay postharvest senescence mainly by slowing ethylene biosynthesis and respiration, reducing oxidative damage, and inhibiting tissue softening. In tomatoes, Yu et al. [104] found that L-Arg slowed coloration and ripening progression. This effect was associated with lower ethylene production due to inhibition of 1-aminocyclopropane-1-carboxylic acid synthase (ACS) and 1-aminocyclopropane-1-carboxylic acid oxidase (ACO) activities and suppressed expression of *SlACS2*/*4* and *SlACO1* genes. In papaya fruit, Arg spraying also delayed ripening, reflected by reduced respiration and ethylene production, slower peel chlorophyll loss, lower weight loss, better maintenance of firmness and nutritional indicators during ambient storage [105]. In sweet cherry fruit, Rehman et al. [106] demonstrated that L-Arg treatment delayed senescence primarily by maintaining cell membrane stability and redox balance. L-Arg decreased H_2_O_2_ and MDA contents, enhanced antioxidant enzymes (SOD, POD, CAT) activities, and suppressed the activities of cell wall degradation enzymes (including cellulase, polygalacturonase, and pectin methylesterase).

### 4.4. Application and Mechanism of Cys in Preservation of Fruits and Vegetables

#### 4.4.1. Disease Control and Mechanisms

Cys controls postharvest diseases in fruits and vegetables through two mechanisms: direct anti-fungal activity and induction of host-mediated resistance. Qu et al. [107] demonstrated that L-Cys reduced conidial germination and mycelial growth, thereby alleviating *Alternaria alternata*-induced black spot disease in pear fruit. This inhibitory effect was associated with impaired pathogen cell membrane function, manifested as increased intercellular leakage and elevated lipid peroxidation. At the molecular level, L-Cys down-regulated genes, including β-1,3-glucan and chitin metabolism, ergosterol and phosphatidylcholine synthesis, and DHN-melanin formation, while up-regulating *GLU* genes. In plum fruit, 100 mg/L L-Cys treatment could inhibit the incidence of brown rot caused by *Monilinia fructicola*, primarily via the enhancement of host defense responses. This effect of L-Cys was due to its role in activating the pentose phosphate pathway and enhancing antioxidant capacity, including higher levels of AsA and GSH as well as higher APX, glutathione reductase (GR), monodehydroascorbate reductase (MDHAR), and dehydroascorbate reductase (DHAR) activities [108].

#### 4.4.2. Quality Maintenance and Mechanisms

Cys primarily maintains the postharvest quality of fruits and vegetables by inhibiting browning, maintaining redox balance, and membrane integrity. In fresh-cut lotus root slices, Gouda et al. [109] demonstrated that L-Cys acted as a positive role in anti-browning by intercepting quinones produced from phenolic oxidation and limiting pigment formation. When combined with citric acid and alginate coating films, it further retained quality by reducing browning and slowing microbial spoilage. This composite coating not only increased ROS scavenging capacity and reduced the accumulation of O_2_·^−^ and H_2_O_2_ but also inhibited the activities of PAL, POD, and enzymes involved in membrane lipid degradation. Cheng et al. [110] reported that combined UV-C and L-Cys treatment altered PPO conformation and promoted enzyme aggregation, thereby limiting access to the active site. Molecular docking further indicated that the binding of L-Cys to PPO through hydrogen bonding and ionic interactions contributes to reducing enzymatic browning in postharvest Lanzhou lily bulbs. In fresh-cut asparagus lettuce, Yu et al. [111] further demonstrated a synergistic combined strategy in which AsA and L-Cys were incorporated into a carboxymethyl cellulose (CMC) coating. This combined treatment showed a more pronounced effect than the single treatments, delaying color deterioration by suppressing PPO/POD and chlorophyll-degrading enzymes, maintaining higher antioxidant capacity, and reducing ROS accumulation. Thus, current evidence points to a possible advantage of Cys in integrated preservation systems, although direct comparisons with standalone treatment remain limited.

#### 4.4.3. Delaying Senescence and Mechanisms

Exogenous Cys can delay postharvest senescence by maintaining redox homeostasis and partly through its role as a precursor of endogenous H_2_S. In Chinese flowering cabbage, Gan et al. [112] found that L-Cys slowed leaf yellowing by down-regulating the expression of chlorophyll catabolic and senescence-associated genes, while maintaining ROS balance via higher antioxidant capacity, manifested as the activation of SOD, POD, CAT, and AsA–GSH cycle. Importantly, L-Cys increased endogenous H_2_S through enhanced L-Cys desulfhydrase activity and up-regulation of H_2_S-biosynthetic genes, linking Cys metabolism to anti-senescence signaling. In leafy vegetables, AI Ubeed et al. [113] confirmed that D-Cys and L-Cys treatments similarly delayed senescence by reducing respiration rates and ethylene biosynthesis, which was consistent with the known protective effects of H_2_S during storage. In tomato fruit, L-Cys delayed ripening by reducing ethylene biosynthesis, resulting from lower ACS and ACO activity and expression of key ethylene pathway genes, thereby delaying ripening-associated quality deterioration [104]. Compared with L-Arg, L-Cys appeared to act more on maintaining flavor-related quality by preserving higher titratable acidity and soluble sugar contents, whereas its delay effect was concentrated mainly at the early ripening stage. Although Cys also suppressed ACS/ACO activity and *SlACS2*/*4* and *SlACO1* expression, its influence on coloration and senescence was less persistent than that of L-Arg, which showed a more sustained inhibition of ripening-associated ethylene biosynthesis.

### 4.5. Application and Mechanism of Pro in the Preservation of Fruits and Vegetables

#### 4.5.1. Chilling Injury Alleviation and Mechanisms

At present, direct postharvest application of Pro has been relatively less studied, and the available evidence suggests that its protective effect is exerted mainly through osmotic adjustment, membrane stabilization, and reinforcement of antioxidant/redox homeostasis under cold stress. In pomegranates, Molaei et al. [17] found that postharvest immersion in 20 mM Pro for 20 min can significantly alleviate chilling injury. Mechanistically, exogenous Pro reduced electrolyte leakage and MDA accumulation, retained higher endogenous Pro levels, enhanced DPPH scavenging capacity and the activities of SOD, CAT, and APX, and maintained higher ascorbic acid content. At the same time, it promoted PAL activity and the accumulation of total phenols, flavonoids, and anthocyanins while suppressing PPO activity, indicating that exogenous Pro alleviated chilling injury in pomegranate through coordinated reinforcement of antioxidant capacity, phenylpropanoid metabolism, and membrane stability. In peach fruit, Gohari et al. [114] discovered through the respective applications of Pro and L-Cys that the 15 mM Pro treatment reduced H_2_O_2_, MDA, and electrolyte leakage, enhanced SOD, CAT, APX, and PAL activities, endogenous Pro accumulation, and flavonoid levels, indicating a broader stress-protective role under cold storage. By contrast, although 0.4% L-Cys also reduced internal browning, its effect was more closely linked to anti-browning and eating-quality maintenance, as shown by better retention of titratable acidity, firmness, and total phenols. Thus, Pro appeared mainly to strengthen osmotic and antioxidant protection, whereas L-Cys in the compared studies contributed more directly to browning suppression and quality retention.

#### 4.5.2. Quality Maintenance and Mechanisms

Beyond chilling tolerance, Pro has been repeatedly linked with postharvest quality maintenance. In fresh-cut potatoes, exogenous Pro pre-treatment inhibited enzymatic browning by reducing PPO activity and total phenol content, promoting endogenous Pro accumulation, reshaping free amino acid profiles, and enhancing antioxidant capacity, thereby extending shelf life under cold storage [115]. In cassavas, Tang et al. [116] found that Pro reduced postharvest physiological deterioration by reducing H_2_O_2_ content and enhancing the activity of key antioxidant enzymes (CAT, SOD, and APX), thereby alleviating oxidative damage and extending shelf life. A combined preservation strategy was reported in strawberry fruit. Bahmani et al. [117] evaluated Pro together with chitosan-based coatings and found that the Pro-coated chitosan nanoparticle treatment (CTS–Pro NPs), rather than Pro alone, showed the clearest preservation effect. This combined treatment reduced H_2_O_2_ and MDA accumulation, decreased decay and weight loss, and improved ascorbic acid, total soluble solids, total phenols, and antioxidant capacity, suggesting that Pro can also contribute to quality maintenance when incorporated into an edible coating system that strengthens surface protection and antioxidant defense.

### 4.6. Application and Mechanism of Phe in the Preservation of Fruits and Vegetables

#### 4.6.1. Disease Control and Mechanisms

Phe has been reported to inhibit postharvest decay through activation of fruit defense responses, either as a standalone elicitor or as an efficacy-enhancing additive in combined treatments. In mangos, Patel et al. [118] used a multi-omics approach to demonstrate that Phe-induced resistance was associated with the up-regulation of defense-related genes, including Ca^2+^ signaling (*CaM*, *CML*, *CDPK*, *Rboh*), mitogen-activated protein kinase (MAPK) cascades (*MEKK 1*, *MAPK 3*, *MKS 1*), WRKY transcription factors (*WRKY33*, *WRKY11*), and enhanced flavonoid and anthocyanin accumulation (such as *PAL*, *4CL*, *COMT*). Furthermore, phenolic extract from the peel of treated fruit inhibited multiple fungi in vitro. In pears, 2.0 mM Phe reduced the diameter of lesions caused by *Alternaria alternata* via increasing the activity of PAL, 4CL, C4H, POD, and PPO, and activating the CaM/CDPK–MAPK–WRKY transcriptional cascade [119]. In integrated preservation systems, Phe also performed effectively as a functional additive rather than a standalone treatment. In citrus, addition of 1 g/L Phe significantly enhanced the efficacy of sodium dehydroacetate in controlling green mold and natural decay, and this improvement was associated with lower H_2_O_2_, O_2_·^−^, and MDA accumulation, stronger antioxidant activity, and higher PAL, C4H, and 4CL activities with increased phenolic and flavonoid accumulation [120].

#### 4.6.2. Chilling Injury Alleviation and Mechanisms

Phe mitigates chilling injury mainly by retaining redox balance and protecting the cell membrane, as well as modulating phenylpropanoid metabolism and texture changes. In plums, Sogvar et al. [121] showed that 7.5 mM Phe increased PAL activity and promoted the accumulation of phenolics, flavonoids, anthocyanins, and AsA, while enhancing SOD, CAT, and APX activity, thereby preserving cell membrane integrity and reducing chilling injury symptoms. In nectarines and tomatoes, Liu et al. [122,123] provided multi-omics evidence that Phe up-regulated key genes in phenylpropanoid metabolism (*PAL*, *C4H*, *4CL*) and enhanced antioxidant capacity, which in turn repressed oxidative damage under chilling stress. At the same time, Phe tended to suppress activities related to cell-wall disassembly, slowing softening during chilling injury development. Xie et al. [124] showed that preharvest Phe alleviated chilling injury in muskmelons by maintaining ROS homeostasis (higher AsA, GSH, and antioxidant capacity, lower O_2_·^−^ and H_2_O_2_) and improving membrane lipid integrity, including a higher degree of unsaturation and reduced phospholipid degradation, which together decreased membrane lipid peroxidation under cold stress.

#### 4.6.3. Quality Maintenance and Mechanisms

In addition, Phe can maintain postharvest quality mainly by preserving texture, visual appearance, and metabolic stability. In pears, Wang et al. [125] demonstrated that postharvest Phe maintained fruit firmness and soluble solids content by inhibiting key tricarboxylic acid cycle enzyme activity and associated gene expression. More specifically, Phe helped maintain peel color and overall visual quality by suppressing chlorophyll catabolism-related genes and lowering respiratory consumption during storage. Manda-Hakki and Hassanpour [126] reported that 4–8 mM L-Phe reduced quality deterioration in strawberries by increasing total phenolics, total flavonoids, anthocyanins, and the activities of CAT, SOD, APX, POD, and PAL under cold storage. In fresh-cut muskmelons, Wang et al. [127] found that L-Phe improved texture and slowed deterioration by enhancing NOX, SOD, CAT, POD, and AsA–GSH cycle functions while increasing AsA, GSH, and phenolics. This indicated quality preservation through regulating ROS homeostasis. In tomatoes, Phe delayed softening development by reducing pectate lyase, β-galactosidase, and cellulase activity, which alleviated cell-wall disassembly. Phe also altered hormonal signaling, increased melatonin levels, and regulated the expression of genes related to various hormone signaling pathways (down-regulate *ACO*, *ABI5*, *SARF4*, up-regulate *SlGRAS10*) [123].

### 4.7. Applications and Mechanisms of Amino Acid Derivatives in Preservation (ε-Polylysine, Betaine)

ε-PL and GB represent two distinct but complementary amino acid-derivative strategies in postharvest preservation. ε-PL is a microbial peptide preservative with direct anti-fungal activity and can also induce host defense responses. ε-PL can inhibit pathogens by disrupting fungal hyphae and cell membranes, and it also activates fruit defense responses in treated tissues. In citrus, Zhang et al. [128] demonstrated that ε-PL enhanced disease resistance by altering metabolism: amino acid and phenylpropanoid metabolism were activated, the activity of phenylpropanoid metabolism-related enzymes (PAL, C4H, 4CL) increased, and the accumulation of phenolic acids and flavonoids (such as ferulic acid, chlorogenic acid, and catechin) increased, thereby improving resistance to *Penicillium* species. However, evidence from fresh-cut produce suggests that ε-PL often performs better as part of a multi-hurdle system rather than as a stand-alone treatment. In vacuum-packed lettuce, ε-PL combined with UV-C better maintained color and texture and suppressed aerobic bacteria [129]. In fresh-cut jackfruit, Zeng et al. [130] found that ε-PL combined with nisin significantly inhibited microbial proliferation, reduced respiration and browning enzyme activity, decreased H_2_O_2_ and MDA levels, and ultimately maintained a higher sensory quality.

GB is a quaternary ammonium amino acid derivative that functions as an osmoprotectant and supports antioxidant regulation in plants. In peach fruit, 10 mM GB soaking reduced internal browning, electrolyte leakage, and MDA. Its protective effect is associated with enhanced antioxidant metabolism through the PpbHLH130-mediated activation of *PpAPX*/*PpPOD*, accompanied by the up-regulation of Pro- and polyamine-related genes linked to PpHsfA2a. Similarly, in bananas, Niaz et al. [131] demonstrated that GB (often combined with a heat shock) reduced the chilling injury index by nearly half and preserved firmness by improving phenolic content and activities of PAL, C4H, and 4CL, while balancing sucrose, glucose, and fructose levels. Mahmoudi et al. [132] effectively reduced EL, MDA, and H_2_O_2_ content by applying glycine betaine coated chitosan nanoparticles (CTS-GB NPs) treatment to plums, thereby significantly alleviating chilling injury. Overall, current evidence suggests that ε-PL is better viewed as a natural antimicrobial component that can also prime defense metabolism, whereas GB primarily mitigates chilling injury through membrane stabilization, antioxidant regulation, and osmotic/metabolic adjustment.

The following three tables (as shown in Table 1, Table 2 and Table 3) summarize the application methods, concentrations, and primary effects of major amino acids when treating various crops to address issues such as disease, chilling injury, quality deterioration, and senescence, providing a more intuitive comparison of amino acid applications in fruit and vegetable preservation. As shown in the tables, immersion is currently the most common method for treating post-harvest produce with amino acids, followed by spraying and injection. For the same crop, such as fresh-cut potatoes, the concentrations used for different amino acids (such as GABA and Glu) are different; their mechanisms of action also differ: GABA primarily enhances antioxidant defenses to mitigate oxidative damage, while Glu directly inhibits PPO activity by lowering pH, chelating Cu^2+^, and binding to the PPO active site. Most available studies still focus on single-crop experiments conducted under specific conditions. Therefore, although existing results indicate that amino acids have great potential for postharvest preservation, their effectiveness remains clearly dependent on crop type and environmental conditions.

## 5. Conclusions

In summary, amino acids have a significant influence on the preservation of postharvest fruits and vegetables. Based on the literature synthesized in this review, GABA is the amino acid with the most consistent supporting evidence, demonstrating repeatedly observed effects in alleviating chilling injury, enhancing disease resistance, and maintaining quality. Phe and Arg also show relatively strong support. In contrast, the evidence for Glu and Cys, and particularly for Pro, remains relatively limited, often being commodity-specific or based on a smaller number of experimental systems. At the phenotypic level, amino acid treatments typically reduce the incidence of decay, mitigate softening, browning, and chilling injury, and help maintain color, firmness, and nutritional attributes. At the metabolic level, amino acids enhance antioxidant capacity, maintain cell membrane and cell wall integrity, and regulate osmotic and energy balance by integrating multiple pathways, including ROS metabolism, phenylpropanoid metabolism, the AsA-GSH cycle, the GABA shunt, and Pro/polyamine metabolism. At the molecular level, amino acids activate signaling pathways such as Ca^2+^ signaling, MAPK cascades, and hormone signaling (ethylene, JA, ABA), upregulating genes for antioxidant enzymes (such as SOD, CAT, APX), phenylpropanoid metabolism (PAL, C4H, 4CL), defense-related genes (such as *PR*, *CHI*), and metabolic regulatory transcription factors, thereby achieving systemic regulation from signal perception to defense response. From a postharvest management perspective, these effects suggest that amino acids may have practical value as potential green tools for reducing storage losses and helping maintain market quality during handling, storage, and distribution, subject to validation of cost, formulation stability, regulatory compatibility, and commercial-scale performance. They may be particularly useful as components of integrated preservation systems, where they can complement existing cold storage, coating, or antimicrobial strategies to improve efficacy while reducing dependence on conventional chemical inputs.

## 6. Future Perspectives

Future work should move beyond single-commodity proof-of-concept studies toward translational validation across the postharvest chain. Based on the evidence synthesized in this review, commodity-specific protocols should be established for each candidate by optimizing concentration, treatment timing, tissue status, maturity stage, storage temperature, atmospheric conditions, and application mode under commercially relevant storage, transport, and shelf-life scenarios. The next phase of research should also include evaluations of formulation stability, purity, cost-effectiveness, and regulatory compatibility, because these factors will determine whether amino acid treatments can move beyond laboratory efficacy to practical postharvest management.

A second priority is to connect the mechanism more directly with practical use conditions. Future studies should distinguish clearly between signaling or induced-resistance effects, which are often dependent on developmental stage and the interval between treatment and stress exposure, and direct physicochemical effects, such as pH-mediated PPO inhibition, metal chelation, membrane stabilization, or short-term redox buffering. This distinction is essential for predicting treatment reliability, defining the optimal timing of application, and deciding whether immersion, spraying, coating, or other delivery systems are most suitable in packinghouse operations. Multi-omics approaches will be more informative when integrated with physiological, microbiological, sensory, and shelf-life assessments to identify biomarkers that predict treatment efficacy, thereby enabling amino acid-based strategies to be rationally integrated into combined preservation systems, including cold storage, edible coatings, controlled atmospheres, and biological control.

## Figures and Tables

**Table 1:** Methods of Controlling Plant Diseases with Amino Acids.

Amino Acids	Types of Fruits and Vegetables	Treated Conditions	Functions	References
GABA	Cherry tomatoes	Hypotonic treatment with 10 mM GABA.	Enhanced *Botrytis* Resistance.	[72]
	Mangoes	2 mM GABA; immersion.	Enhanced *Colletotrichum gloeosporioides* resistance.	[133]
	*Mesembryanthemum crystallinum* L.	200 mg/L GABA; immersion.	Reduced rot caused by *Fusarium*.	[73]
Glu	Pears	Wound injection with 1.00 mM L-Glu.	Inhibited spore germination.	[15]
	Apples	0.1 g/L L-Glu; immersion.	Inhibited the expansion of blue mold disease.	[87]
	Tomatoes	100 ppm L-Glu; immersion.	Reduced rot caused by gray mold.	[85]
Arg	Winter jujubes	200 μM Arg; immersion.	Reduced decay caused by Alternaria alternata.	[94]
	Strawberries	1 mM Arg; immersion.	Reduced the incidence of decay and decay index.	[96]
	Blueberries	1 mM L-Arg; spraying.	Reduced Alternaria fruit rot.	[95]
Cys	Plums	100 mg/L L-Cys; Inoculation at the wound site.	Reduced the severity of brown rot.	[108]
	Pears	1 g/L L-Cys; immersion.	Effectively controlled and prevented black spot disease.	[107]
Phe	Mangoes	8 mM Phe; immersion.	Induced disease resistance.	[118]
	Pears	2 mM Phe; immersion.	Reduced the diameter of *Alternaria* leaf spots.	[119]
	Citruses	1 g/L Phe; Combined with sodium dehydroacetate.	Enhanced the control efficiency of natural decay diseases.	[120]

**Table 2:** Methods for Alleviating Chilling Injury (CI) with Amino Acids.

Amino Acids	Types of Fruits and Vegetables	Treated Conditions	Functions	References
GABA	Peaches	5 mM GABA; immersion 20 min.	Maintained higher TSS and extractable juice.	[78]
	Chinese olives	1 mM GABA; immersion 10 min.	CI index decreased by 51.5%. MDA decreased by 32.4% (100 days).	[75]
	Orlando tangelos	5 mM GABA; immersion 10 min.	CI index and MDA decreased; total phenols, flavonoids, and CAT/POD increased.	[80]
Glu	Prunes	0.1 g/L L-Glu; immersion 10 min.	Internal browning index decreased by 52.86%; EL decreased by 16.10%; MDA decreased by 10.67%; PPO decreased by 18.85% (48 d).	[88]
Arg	Pomegranates	1 mM Arg; preharvest spray + postharvest immersion.	Husk browning decreased; EL, MDA, and H_2_O_2_ decreased.	[98]
	Plums	0.5% CTS–Arg NPs; coating	CI index decreased by 87.3% (40 days); EL decreased by 42.05%; MDA decreased by 39.1%.	[100]
Pro	Pomegranates	20 mM Pro; immersion 20 min.	CI decreased by 89.1% (90 + 3 d), EL decreased by 19.5%, and MDA decreased by 42.6%.	[17]
Phe	Tomatoes	8 mM Phe; immersion 15 min.	CI index decreased; MDA decreased; AsA, total phenols, flavonoids, and antioxidant enzyme activity increased.	[123]
	Nectarines	8 mM Phe; immersion 15 min.	CI index decreased; extractable juice decreased; MDA decreased.	[122]
	Muskmelons	8 mM Phe; preharvest spraying.	CI index decreased by 26.5%; MDA decreased by 20.2% (28 d).	[16]

**Table 3:** Methods for Amino Acids to Maintain Quality and Delay Aging.

Amino Acids	Types of Fruits and Vegetables	Treated Conditions	Functions	References
GABA	Fresh-cut potatoes	20 g/L GABA; immersion 10 min.	Browning development decreased.	[134]
	Fresh-cut stem lettuces	10 mM GABA; immersion 5 min.	Browning degree, PPO activity, MDA content, and electrolyte leakage decreased; chlorophyll and ascorbic acid contents increased.	[83]
	Sweet cherries	50 mM GABA; preharvest foliar spray.	Firmness, TSS, TA, total phenolics, anthocyanins, and antioxidant enzyme activities increased.	[135]
	Strawberries	10 mM GABA; immersion 15 min.	Weight loss, ROS, and MDA decreased; total soluble sugar, titratable acid, SOD, CAT, anthocyanins, and flavonoids increased.	[136]
Glu	Pears	0.5 μM L-Glu; immersion 10 min.	Ethylene release, respiratory intensity decreased; ascorbic acid, soluble solids, and soluble sugars increased.	[90]
	Fresh-cut carrots	0.50 g/L Glu; immersion 10 min.	Degradation of total carotenoids decreased; antioxidant capacity increased.	[19]
	Fresh-cut potatoes	15 g/L Glu; immersion 4 min.	Browning decreased; PPO activity and total phenolic content decreased.	[92]
Arg	Papayas	25 mg/L Arg; spraying.	Respiration rate, ethylene production, and chlorophyll loss decreased; firmness was better maintained; ripening was delayed.	[105]
	Fresh-cut pears	200 mM Arg; dipping for 5 min.	Browning index and weight loss decreased; firmness and TSS were not significantly affected.	[103]
	Persimmons	1 mM L-Arg; immersion 10 min.	Weight loss, decay incidence, electrolyte leakage, MDA decreased; ascorbic acid, antioxidant activity, and total phenolics increased.	[102]
Cys	Chinese flowering cabbages	0.5 g/L L-Cys; immersion 3 min.	Leaf yellowing and chlorophyll degradation decreased; soluble sugar and total flavonoids were better maintained; senescence was delayed.	[112]
	Goji fruits	0.05% Cys; immersion 5 min.	Weight loss and decay ratio decreased; TSS, total phenolics, and ascorbic acid, total GSH increased.	[137]
	Pears	0.1 mM L-Cys; immersion 30 min.	Weight loss, MDA, pH, TSS, and PPO activity decreased; TA, total phenols, total flavonoids, ascorbic acid, PAL, GR, and APX activities increased	[126]
Pro	Cherry tomatoes	1 g/L Pro; preharvest foliar spray.	H_2_O_2_ decreased; SOD activity increased; postharvest quality deterioration was delayed.	[12]
Phe	Strawberries	4 mM Phe; immersion.	Weight loss and MDA decreased; total phenolics, total flavonoids, anthocyanins, and antioxidant capacity increased.	[126]
	Pears	2 mM Phe; immersion 10 min.	Firmness and soluble solids were better maintained; TCA-related metabolism and chlorophyll degradation decreased.	[125]

## Data Availability

No new data were created or analyzed in this study.
